# Effect of Pesticide Exposure on Immunological, Hematological and Biochemical Parameters in Thai Orchid Farmers—A Cross-Sectional Study

**DOI:** 10.3390/ijerph120605846

**Published:** 2015-05-27

**Authors:** Soraya Aroonvilairat, Wannapa Kespichayawattana, Thiwaree Sornprachum, Papada Chaisuriya, Taweeratana Siwadune, Kavi Ratanabanangkoon

**Affiliations:** 1 Center of Excellence on Environmental Health and Toxicology (EHT), and Laboratory of Immunology, Chulabhorn Research Institute, Kampangpetch 6 Road, Lak-si, Bangkok 10210, Thailand; E-Mails: saroonv@hotmail.com (S.A.); wannapa@cri.or.th (W.K.); thiwaree@cri.or.th (T.S.); papada@cri.or.th (P.C.); 2 Department of Mathematics, Faculty of Science, Mahidol University, Rama VI Road, Ratchathewi, Bangkok 10400, Thailand; E-Mail: taweeratana.siw@mahidol.ac.th; 3 Chulabhorn Graduate Institute, Kampangpetch 6 Road, Lak-si, Bangkok 10210, Thailand

**Keywords:** pesticide exposure, immunological and biochemical alterations, orchid farmers, cross-sectional study

## Abstract

Various studies have found that many Thai orchid farmers used excessive amounts of pesticides without proper protective gear, but no toxicological study has been made. This cross-sectional study aimed to evaluate the immunological, hematological and biochemical statuses of these farmers. Sixty four orchid farmers and 60 controls were studied. Plasma cholinesterase activity, the percentage and absolute number of B lymphocytes (CD19^+^) were significantly lower in the farmers group (3966.32 ± 1165.48 U/L, 11.61 ± 4.09% and 312.26 ± 164.83 cells/mm^3^, respectively) as compared to those of controls (5048.85 ± 1139.40 U/L, 14.32 ± 4.23%, 420.34 ± 195.18 cells/mm^3^, respectively). There was a statistically significant higher level of serum IgE among the orchid farmers (0.031 ± 0.011 mg/dL *vs.* 0.018 ± 0.007 mg/dL) but not IgG, IgA and IgM, levels. Serum lysozyme level, lymphocyte proliferative responses to mitogens, hematological parameters and kidney function test, were not significantly different between the two groups. The liver function profiles showed significantly lower levels of albumin and serum protein in the farmer group. Thus frequent pesticide exposure resulted in subtle changes of some biological parameters. These changes, though may not be clinically significant, strongly indicated that caution in handing pesticides by these farmers is warranted.

## 1. Introduction

Pesticides are extensively used in agriculture in developing countries. In Thailand, the amount of imported pesticides has escalated continuously from 78,644 tons in 2005 to 134,480 tons in 2012 [1]. Studies on agricultural workers in Thailand and many developing countries have repeatedly shown that farmers often use cocktails of pesticides with different mixtures over different periods of time, and usually at higher concentrations and frequency than recommended [2,3], possibly due to frequent adulteration which included substandard products with low percentages of active ingredients and counterfeit agrochemicals [4].

Orchids are an important agricultural product of Thailand, with an export value of hundreds of millions of US dollars. To scale up production and to reduce labor costs, farmers use increased amounts of fertilizers and pesticides. Reports using questionnaires and fingertip blood tests for butyrylcholinesterase, strongly suggested that orchid farmers improperly handle and dispose of chemical solid waste and were most likely exposed to the pesticides [2], however, there has been no toxicological laboratory study on these Thai farmers.

Chronic low-level exposure to pesticides is associated with serious health problems including metabolism impairment, neurotoxicity, carcinogenicity, reproductive and endocrine disruption as well as immune dysfunctions [5,6,7]. Karami-Mohajeri and Abdollahi [6], for example, in a systematic review found that organophosphates (OP) and carbamates (CB) impair the metabolism of carbohydrates, fats and protein through the inhibition of AChE or affecting target organs directly.

Immunomodulation of chlorpyrifos, an organophosphate (OP) insecticide, has been reported to increase in CD26 cells and multiorgan autoantibodies but decrease in CD5^+^ cells and in the mitogenesis response to phytohemagglutinin and concanavalin A [8,9]. Exposure to the synthetic pyrethroid cypermethrin, tended to lower the immunoglobulins (IgG, IgM, IgA), the complement components C3c and C4 and the acute phase protein α-acid glycoprotein (AAG), and the lymphocyte subpopulations CD3^+^, CD4^+^ and CD20^+^ after pest control operation [10]. Steerenberg *et al.* [11] found increased complement and IgG4 levels, but decreased IgA in European pesticide workers in agriculture who were exposed to a mixture of pesticides including ethylenebisdithiocarbamate (EBDC) fungicides. Chemical plant workers chronically exposed to pesticide dusts including captan and carbendazim showed disturbances in humoral and cellular immunities [12]. Garg *et al.* [13] showed that chronic exposure to small amount of synthetic pyrethroid, organophosphate and chlorinated pesticides lead to deleterious effects on the metabolism and immune system of birds. Pesticides have also been shown by Chatterjee *et al.* [14,15] to induce marrow toxicity and effects on marrow cell population and on hematopoietic stroma that ultimately could lead to the formation of a degenerative disease like aplastic anemia. These studies indicated that pesticide exposures could seriously alter various parameters of the biological systems. The present study assessed the immunological, hematological and biochemical status of Thai orchid farmers who were in frequent contact with pesticides.

## 2. Methods

### 2.1. Study Participants & Areas

This cross-sectional study was conducted at the intense orchid cultivation areas in Thailand in Nakhonpathom and Samutsakhon provinces. One hundred and twenty-four healthy adults, aged of 20–60 years, participated in this study. Eligible subjects were 64 farmers (30 men, 34 women) who had been working and exposed to pesticides in orchid farms for more than 3 months. Their age and gender matched controls (60) were living in the same districts and had other occupations unrelated to farm work or orchid production (33 men, 27 women). Since the farms were quite far apart, many of them were accessible only by motorcycles; the participants were requested by the provincial medical officers to show up to complete a questionnaire interview and blood donation. The interview was carried out on the day when the blood samples were collected. On the pesticides used, the interviewers cited the names of pesticides and showed the pictures of popular brands sold in the areas to the orchid farmers and asked which chemicals they had used in the past 3 months. Information obtained from questionnaires included personal socio-demographic information, agricultural activities and pesticide application as well as self-reported health status and daily lifestyle (smoking habit, medication, alcohol consumption). Subjects having recent severe trauma or surgery or infection and under antibiotic or steroid treatment, diabetes, obesity, and other chronic or immunologic conditions (asthma, allergic to food, pollen or medicine), including pregnant or lactating individual were excluded. Since almost all male farmers and controls were smokers and frequent alcohol drinkers so they were included in this study.

The research protocol was reviewed and approved by the Institutional Ethical Committee of the Chulabhorn Research Institute in agreement with the Declaration of Helsinki for International Health Research (Protocol No. 002/2008). All participants signed informed consent forms prior to enrollment in the study.

### 2.2. Sample Collection

All blood samples were collected by venipuncture during March to April in a total of six field trips. The blood samples (10 mL from each subject) were immediately kept in an ice-cold chamber, transported and delivered to the laboratory for processing within 6 h of blood donation.

### 2.3. Measurement of Plasma and Erythrocyte Cholinesterases

Plasma and erythrocytes were separated by centrifugation (800 × g, 10 min at 25 °C) from whole blood samples containing EDTA as an anticoagulant. Cholinesterase activities in plasma and in red blood cells were analyzed by the method of Ellman *et al.* [16] with the use of 5,5’-dithiobis-(2-nitrobenzoic) acid (DTNB) as a chromagen. Briefly, packed red blood cells were lysed by suspending in 10 volumes of distilled water and the erythrocyte acetylcholinesterase reaction with acetylthiocholine iodide substrate was measured by the formation rate of 5-mercapto-2-nitrobenzoate at room temperature. The changes in absorbance 405 nm in 30 sec were calculated and expressed as unit/L. Plasma butyrylcholinesterase activity was determined by measuring the rate of butyrylthiocholine hydrolysis in the same way as that of red blood cells cholinesterase activity.

### 2.4. Lymphocyte Subpopulations

Whole blood samples containing EDTA as an anti-coagulant were stained with fluorescein isothiocyanate (FITC) or phycoerythrin (PE) conjugated monoclonal antibodies according to the manufacturer’s recommendation. Monoclonal antibodies (BD Biosciences, San Jose, CA, USA) against CD3, CD4, CD8, CD19 and CD16/CD56 were used. The isotype controls using FITC-labeled IgG1 clone X 40 and PE-labeled IgG2a clone X 39 were included in the assay of each blood sample. Following incubation of the whole blood with fluorescent-conjugated monoclonal antibodies, the red blood cells were lysed with fluorescence activated cell sorter (FACS) lysing solution (BD Biosciences), washed with 0.1% sodium azide in PBS, fixed with 1% paraformaldehyde in PBS and kept protected from light at 4 °C. The cells were analyzed by a flow cytometer (FACS Calibur, BD Biosciences) within 24 h of staining. The 10,000 events were acquired and the lymphocyte population was gated on the basis of forward and side scatter signals.

### 2.5. Serum Immunoglobulins

The total serum concentrations of IgA, IgE, IgG and IgM were measured using a sandwich ELISA commercial kit (Bethyl Laboratories, Inc., Montgomery, TX, USA) with appropriately diluted samples in accordance with the manufacturer’s protocol. The average sample dilutions for IgA, IgG, IgM, IgE analysis were about 1: 150,000; 1:50,000; 1:50,000; 1:2, respectively. The calibration curve for each immunoglobulin class was constructed using Human Reference Serum supplied in the kit. Optical density (OD) was read at 450 nm with a Multiskan Ascent microplate reader (Labsystems, Helsinki, Finland). Only lower detection limits of these kits provided by the manufacturer were 0.69 ng/mL for IgG, IgE and 1.03 ng/mL for IgA, IgM. The inter-assay variations of each immunoglobulin in three different serum samples obtained from normal laboratory volunteers were within 10% coefficient of variation (CV).

### 2.6. Lysozyme Assay

The serum samples were separated from clot blood tube by centrifugation (800 × g, 10 min at 25 °C). The activity of serum lysozyme was measured according to the turbidimetric method described by Keller *et al.* [17] with slight modifications. Briefly, aliquots of serum samples were incubated with the substrate suspension of *Micrococcus lysodeikticus* (Sigma-Aldrich, St. Louis, MO, USA) and the lysozyme activity was determined by comparing the absorbance of test serum with the linear regression standard curve of chicken egg white lysozyme (Sigma-Aldrich) at OD450 nm and expressed as U/mL.

### 2.7. Hematological and Blood Biochemical Tests

The blood specimens were transported in an ice box to an accredited standardized laboratory (Bangkok-Pathology Laboratory, Bangkok, Thailand) and were analyzed within 24 h of donation. A complete blood count and differential cell count were performed using venous blood containing EDTA as an anticoagulant. For renal and liver function tests, sera were measured for blood urea nitrogen (BUN), creatinine, total protein, albumin, globulin, aspartate aminotransferase (AST), alanine amino-transferase (ALT) and alkaline phosphatase (ALP).

### 2.8. Mitogen Stimulation Assay

The lymphocyte function assay was carried out within 6 h after blood donation. It was performed by lymphoproliferative responses to mitogens as described by Ng and Zelikoff [18] with slight modifications. The peripheral blood mononuclear cells (PBMC) were obtained from heparinized blood by Ficoll-Paque gradient centrifugation. The viability of PBMC was determined by trypan blue dye exclusion, and all of the PBMC samples had over 95% viable cells. The PBMC (2 × 10^5^ cells) were cultured with medium alone or in the presence of pre-optimized concentrations of mitogens: 5 µg/mL of concanavalin A (Con A; Sigma-Aldrich) or 0.1 µg/mL of pokeweed mitogen (PWM; Sigma-Aldrich) for 96 h at 37 °C in a humidified 5% CO_2_ incubator. Another set of triplicate wells containing medium alone without cells was included as medium controls. The blastogenesis of PBMC was then evaluated by 3-(4,5-dimethylthiazol-2-yl)-2,5-diphenyltetrazolium bromide; (MTT) assay and measured at OD595 nm. The stimulation index (SI) for each mitogen was calculated as:

$$\text{SI =}\frac{\text{OD of mitogen stimulated cells}-\text{OD of medium alone}}{\text{OD of unstimulated cells}-\text{OD of medium alone}}$$

### 2.9. Statistical Analysis

Statistical analyses were performed with the SPSS statistical package version 15 (SPSS Inc., Chicago, IL, USA). The categorical variables were compared by the Chi-square or Fisher’s exact test. Mean differences of all parameters in blood were tested by the Student’s *t* test. One-way ANOVA and multiple comparisons Dunnett’s test were used to compare ChE activities in each farmer subgroups. The values of AST, ALT, the CD8^+^ cell numbers, serum IgE and stimulation index to Con A had been log-transformed and were expressed as the geometric mean. A *p*-value of 0.05 was considered statistically significant.

## 3. Results 

### 3.1. Characteristics of the Study Population

Table 1 shows a summary of socio-demographic characteristics of the study population. The farmers, most of them (87.5%) were Thai, had been working in the orchid farms ranging from three months to 37 years. They had an average of 10 rai (16,000 sq·m or 3.95 acres) field-size working area. Approximately 80% of the orchid farmers lived in their farmland. All control subjects were Thai and had higher education.

**Table 1:** Descriptive characteristics of orchid farmers (*n* = 64) and control populations (*n* = 60) participated in the study.

Characteristics	Orchid farmer Mean ± S.D.	Control Mean ± S.D.	*p*-Value ^#^
Age (years)	41.97 ± 9.23	38.82 ± 10.38	0.076
**Characteristics**	**n (%)**	**n (%)**	***p*-Values ^§^**
Gender - Men	30 (46.9)	33 (55.0)	0.234
Married	47 (73.4)	38 (63.3)	0.154
Cigarette smoker	8 (12.5)	11 (18.3)	0.257
Alcohol user	15 (23.4)	20 (33.3)	0.153
Ethnicity:			
Thai	56 (87.5)	60 (100)	n/a
Hill tribes	8 (12.5)	0 (0)	
Education:			
Primary school or uneducated	48 (75.0)	18 (30.0)	<0.001
Secondary school or higher	16 (25.0)	42 (70.0)	
Residential area:			
On farmland	53 (82.5)	-	
Outside farmland	11 (17.5)	-	
Duration of working in farm (year) **^a^**	15.65 (0.3–37)	-	
Farm area (rai) **^a^**	10.51 (2–70)	-	

**^#^** Student’s *t* test; **^§^** Fisher’s exact test or χ^2^ test; **^a^** Mean (Min-Max); n/a Not assume.

The main pesticides used by orchid farmers are listed in Table 2. All orchid farmers (100%) used insecticides while 89%–98% of them also used fungicides and/or herbicides. Among insecticides, cypermethrin was the most frequently selected by the farmers (76.6%). However, various organophosphates and carbamates were also used, which altogether accounted for 123.4%. The use of all the insecticides listed in Table 2 amounted to 234.4%. These figures indicated that, not one but a mixture of different insecticides was used in the cocktail, and/or the farmers might use different insecticides during the past 3 months. Information from the farmers indicated that 90.3% of these farmers used a “cocktail” of different chemicals, including different insecticides, in spraying the orchids. Furthermore, the types and concentrations of insecticides in the mixtures might change at any period depending on the pest encountered. Captan (62.5%) and mancozeb (60.9%) were two fungicides with comparable frequency of utilization. Glyphosate (54.7%) was the most commonly employed herbicide.

In this study, most of the farmers (87.5 %) were involved mainly in spraying pesticides. Their major work involved mixing the pesticides, spraying pesticides and fertilizers. Apart from these, most farmers were involved in flower cutting and selling or transporting the flowers to the market. They usually did not have other profession. Over 95% of them applied pesticides at least once a week, on the average every 5 days. During the rainy season, however, most orchid farmers sprayed pesticide cocktails at least twice weekly. It would take a farmer about 8 hours to spray a farm of 16,000 sq·m area. Regarding the use of personal protective equipment (PPE), the sprayers used headgear (77%), nose/mouth cover (62.5%) and shoes (70%). Less than 10% of the farmers wore goggles or rubber gloves, but no farmers reported mixing pesticide cocktails with their bare hands as they previously practiced. The protective equipment including the headgear and nose/mouth cover was mostly made of cotton fabric materials which should not provide adequate protection. Since no farmer wore fully protective PPE, no statistical analysis on those used and did not use the PPE was made. All farmers mentioned the importance of staying upwind during spraying.

**Table 2:** Main pesticides used in orchid farms in the study areas.

Pesticides	Number of Farmers (*n* = 64)(%) ^#^	Chemical Class	Toxicological Classes *
***Insecticide***	***64 (100)***		
Cypermethrin	49 (76.6)	Pyrethroid	Ib
Methomyl	35 (54.7)	Carbamate	Ib
Abamectin	22 (34.4)	Botanical	Not listed
Chlorpyrifos	19 (29.7)	Organophosphate	II
EPN	10 (15.6)	Organophosphate	Ia
Carbosulfan	8 (12.5)	N-Methyl carbamate	II
Omethoate	7 (10.9)	Organophosphate	Ib
***Fungicide***	***63 (98.4)***		
Captan	40 (62.5)	Thiophthalimide	U
Mancozeb	39 (60.9)	Dithiocarbamate, inorganic zinc	U
Carbendazim	12 (18.8)	Benzimidazole	U
***Herbicide***	***57 (89.1)***		
Glyphosate	35 (54.7)	Phosphonoglycine	U
Paraquat	21 (32.8)	Bipyridylium	II

**^#^** Participants could answer multiple responses; * Ia: Extremely hazardous; Ib: Highly hazardous; II: Moderately hazardous; III: Slightly hazardous; U: Unlikely to present acute hazard (WHO classification).

### 3.2. Erythrocytes and Plasma Cholinesterases

To determine whether the orchid farmers had been exposed to OP insecticides, the activities of cholinesterases in red blood cells and in plasma were assayed. The results showed a significant reduction of about 20% in plasma cholinesterase activity in all 64 orchid farmers as compared to that of 60 controls (males: 4213.39 ± 1155.11 *vs.* 5291.48 ± 1191.15 U/L; females: 3748.31 ± 1147.26 *vs.* 4752.30 ± 1016.69, *p <* 0.001). The plasma enzyme levels in farmers (24 males and 28 females) who used mainly organophosphate and/or carbamate insecticides (males: 4110.82 ± 1153.54 U/L; females 3749.76 ± 1206.88 U/L,) were significantly lower than that of controls (*p* < 0.01) (Table 3). The plasma enzyme levels of farmers (6 males and 6 females) who used mainly pyrethroid (PY) insecticides (males: 4623.67 ±1168.45 U/L; females: 3741.56 ± 906.34 U/L) and those of controls were comparable (*p* > 0.05). However, the plasma enzyme levels of these two groups of farmers did not differ significantly. Further analysis of the farmers with different levels of plasma enzyme inhibition was made. Enzyme inhibition was considered moderate with >25%–35% and severe with >35% of ChE inhibition. Among the male OP/CB users, three (12.5%) and five (20.8%) were found to be in the moderate and severe inhibition groups, respectively. The corresponding values for female users were four (14.3%) and five (17.9%), respectively. For the male PY users, the moderate enzyme inhibition was found only one (16.7%) with no severe inhibition. The corresponding values for female users were one (16.7%) and one (16.7%), respectively. The acetylcholinesterases in red blood cells of these groups of farmers were comparable (Table 3). It should be pointed out that almost all (90.3%) of the orchid farmers used mixtures of pesticides including insecticides. Thus, although the farmers indicated that they used mainly pyrethroids but most likely other insecticides e.g., OP and/or CB were also included, and *vice versa*.

**Table 3:** Cholinesterase activities of orchid farmers and controls (Mean ± S.D.)

Enzymes (U/L)	Subjects			Normal Ranges
	OP/CB Using Farmers	PY Using Farmers	Controls	
Plasma ChE				
Male	4110.82 ± 1153.54 ** (*n* = 24)	4623.67 ± 1168.45 (*n* = 6)	5291.48 ± 1191.15 (*n* = 33)	>4900
Female	3749.76 ± 1206.88 ** (*n* = 28)	3741.56 ± 906.34 (*n* = 6)	4752.30 ± 1016.69 (*n* = 27)	>4300
Erythrocyte ChE	7958.55 ± 2280.83 (*n* = 52)	7958.55 ± 2222.82 (*n* = 12)	8137.39 ± 1779.88 (*n* = 60)	8700–11,600

** Significant differences with control group at *p* < 0.01.

### 3.3. Serum Immunoglobulin Levels

The levels of various serum immunoglobulin classes are summarized in Table 4. The concentrations of IgG, IgA and IgM in the orchid farmers and the controls were not significantly different. These levels were within the reference ranges. However, the IgE concentration was significantly higher in the orchid farmers than those of the controls and was over the reference range.

**Table 4:** Levels of serum immunoglobulins in orchid farmers and in controls (Mean ± S.D.).

Immunoglobulin Isotypes (mg/dL)	Orchid Farmers (*n* = 64)	Controls (*n* = 60)	*p*-Values	Reference Ranges ^#^
IgG	1094.27 ± 219.40	1090.46 ± 204.02	0.920	700–1600
IgA	308.49 ± 109.01	331.06 ± 148.23	0.339	70–400
IgM	151.25 ± 63.52	148.00 ± 81.93	0.805	40–230
IgE	0.031 ± 0.011	0.018 ± 0.007	0.009 **	<0.024

** Significant differences with control group at *p* < 0.01; **^#^** The reference ranges in healthy adults from Gonzalez-Quintela *et al.* [19].

### 3.4. Lymphocyte Subpopulations and Functional Study

The enumeration of lymphocyte subsets in orchid farmers showed significant decrease both in the percentages of CD19^+^ cells (Table 5) and in absolute B lymphocytes count (312.26 ± 164.83 *vs.* 420.34 ± 195.18 cells/mm^3^, *p <* 0.001). The percentages of other lymphocyte subpopulations, including CD3^+^, CD4^+^, CD8^+^ and CD16^+^/CD56^+^ were not significantly different among the two groups (Table 5). The functional study of the immune system was assessed by the blastogenesis of lymphocytes following Con A and PWM stimulation. No significant difference in the stimulation index to each mitogen was detected among the farmers and control groups (Table 6).

**Table 5:** Percentages of lymphocyte subpopulations in orchid farmers and in controls (Mean ± S.D.)

Immunophenotypes (%)	Orchid Farmers (*n* = 64)	Controls (*n* = 60)	*p*-Values	Reference Ranges ^#^
CD4^+^	33.82 ± 7.29	33.72 ± 5.81	0.930	24.10–50.70
CD8^+^	31.76 ± 5.97	30.27 ± 4.37	0.282	19.50–44.60 (Female)
	35.91 ± 7.40	35.40 ± 5.33	0.589	17.70–42.70 (Male)
CD4:CD8 ratio	1.07 ± 0.40	1.07 ± 0.33	0.928	0.65–2.49
CD3^+^	66.74 ± 8.15	64.89 ± 6.87	0.349	57.80–82.70 (Female)
	62.0 ± 8.31	61.86 ± 7.12	0.944	46.20–78.90 (Male)
CD19^+^	11.61 ± 4.09	14.32 ± 4.23	*<*0.001 ***	7.70–25.40
CD16^+^/CD56^+^	15.75 ± 7.87	15.77 ± 7.64	0.990	6.40–31.10 (Female)
	19.63 ± 8.27	18.88 ± 6.93	0.698	3.90–38.50 (Male)

*** Significant differences with control group at *p* < 0.001; **^#^** The normal ranges in healthy Thai adults from Webster *et al.* [20].

**Table 6:** The stimulation index (S.I.) of lymphoproliferative responses to mitogens in orchid farmers and in controls (Mean ± S.D.)

Stimulation Index (S.I.)	Orchid Farmers (*n* = 64)	Controls (*n* = 60)	*p*-Values
Concanavalin A (Con A)	2.48 ± 1.53	2.69 ± 1.41	0.256
Pokeweed mitogen (PWM)	1.90 ± 0.76	2.12 ± 0.69	0.095

### 3.5. Serum Lysozyme Levels

Lysozyme has the ability to lyse and kill gram positive bacteria via enzymatic cleavage of peptidoglycan [17]. The measurement of lysozyme activity thus implies the status of non-specific defense of the immune system. There was a modest increase in the serum lysozyme levels in the farmers compared to controls (474.46 ± 105.67 *vs.* 444.43 ± 75.43 U/mL, *p* = 0.081). However, the difference was not statistically significant.

### 3.6. Analysis of Hematological and Biochemical Parameters

For the biochemical analysis of the sera, the BUN and creatinine which are primarily indicators of kidney function did not differ between the two groups (Table 7). The evaluation of serum enzymes and protein factors showed significant differences in the levels of albumin and total protein. The mean concentration of albumin was lower in the orchid farmers (4.55 ± 0.31 g/dL), thus resulting in the lower level of total serum protein (7.59 ± 0.42 g/dL). These values in the control group were 4.70 ± 0.32 and 7.80 ± 0.43 g/dL, respectively (Table 7). Although there were significant differences in these parameters, the mean values were within the reference ranges. The complete and differential blood cell count did not significantly differ between the farmers and the controls (Table 8).

**Table 7:** Levels of serum protein/factors in orchid farmers and in controls (Mean ± S.D.).

Parameters	Orchid Farmers (*n* = 64)		Controls (*n* = 60)		*p*-Values	Normal Ranges
	Mean ± S.D.	% abnormal	Mean ± S.D.	% abnormal		
***Kidney function profiles***						
BUN (mg/dL)	12.64 ± 3.70	3.1	12.43 ± 2.87	1.7	0.729	7–20
Creatinine (mg/dL)	0.86 ± 0.11 (*n* = 34)	2.9	0.82 ± 0.10 (*n* = 27)	3.7	0.106	0.6–1.1 (Female)
	1.09 ± 0.11 (*n* = 30)	0	1.09 ± 0.10 (*n* = 33)	0	0.946	0.9–1.3 (Male)
***Liver function profiles***						
Total protein (g/dL)	7.59 ± 0.42	12.5	7.80 ± 0.43	25.0	0.007 **	6–8
Albumin (g/dL)	4.54 ± 0.31	3.1	4.70 ± 0.32	16.7	0.007 **	3.5–5
Globulin (g/dL)	3.05 ± 0.32	7.8	3.10 ± 0.33	8.3	0.370	1.5–3.5
AST (U/L)	22.45 ± 1.27	3.1	21.36 ± 1.30	3.3	0.278	0–37
ALT (U/L)	17.64 ± 1.74	7.8	20.44 ± 1.91	18.3	0.175	0–40
ALP (U/L)	64.78 ± 16.98	0	71.08 ± 19.30	3.3	0.056	26–117

** Significant differences with control group at *p* < 0.01.

**Table 8:** Hematological profile of orchid farmers and controls (Mean ± S.D.).

Parameters	Orchid Farmers (*n* = 64)		Controls (*n* = 60)		*p*-Values	Normal Ranges
	Mean ± S.D.	% abnormal	Mean ± S.D.	% abnormal		
WBC count (10^3^/mm^3^)	7.13 ± 1.61	1.6	7.33 ± 1.67	3.3	0.516	4–11
*Neutrophil (%)*	53.23 ± 10.30	25.0	51.38 ± 9.54	25.0	0.302	45–75
*Lymphocyte (%)*	37.44 ± 8.69	20.3	39.43 ± 7.48	20.0	0.174	20–45
*Monocyte (%)*	5.19 ± 3.45	20.3	5.40 ± 3.22	18.3	0.499	2–10
*Eosinophil (%)*	4.13 ± 4.13	84.4	3.75 ± 4.56	76.7	0.801	4–6
*Basophil (%)*	0.03 ± 0.18	0	0.00 ± 0.00	0	0.169	0–1
RBC count (10^6^/mm^3^)	4.56 ± 0.60	70.6	4.68 ± 0.51	55.6	0.402	4.5–5 (Female)
	5.01 ± 0.53	26.7	5.18 ± 0.44	9.1	0.162	4.5–6 (Male)
Hemoglobin (g/dL)	12.63 ± 1.88	35.3	13.08 ± 1.29	25.9	0.292	12–16 (Female)
	14.64 ± 1.01	26.7	14.54 ± 1.08	27.3	0.703	14–18 (Male)
Hematocrit (%)	38.07 ± 5.22	35.3	39.50 ± 3.88	11.1	0.238	35–45 (Female)
	43.59 ± 3.08	20.0	43.21 ± 2.55	18.2	0.597	41–51 (Male)
MCV (fL)	85.65 ± 7.72	28.1	84.19 ± 5.54	26.7	0.231	82–96
MCH (pg)	28.57 ± 3.04	32.8	28.10 ± 2.33	30.0	0.338	27–32
MCHC (g/dL)	33.34 ± 1.10	12.5	33.38 ± 1.03	8.3	0.811	32–36
Platelet count (10^3^/mm^3^)	257.73 ± 58.63	7.8	265.92 ± 62.58	1.7	0.454	150–400

## 4. Discussions

There have been many studies suggesting high pesticide exposure in Thai agricultural workers, including orchid planters [2,3,21,22,23,24]. If this is the case, there would be a large number of farmers vulnerable to the toxic effects of pesticides. Therefore it is necessary to investigate this problem and if possible, find ways to prevent and/or remedy the situation.

The present study found reductions in plasma butyrylcholinesterase activity in orchid farmers, suggesting exposure to OPs and/or CMs. Plasma cholinesterase in farmers who used mainly OPs and/or CM insecticides was significantly lower than those of controls, while farmers who used mainly pyrethroids had comparable levels to those of controls. In this study, the average spraying interval was 5 days and the enzyme levels were measured within 4 days after pesticide exposure. Since the recovery of plasma butyrylcholinesterase activity from dimethoate OP inhibition is about 3 days and much longer from fenthion inhibition [25], the observed decrease in the plasma enzyme activity in exposed farmers was likely due to OP. However, because plasma enzyme concentrations are more variable than those measured in erythrocytes [26] and Garabrant *et al.* [27] suggested that a 20% depression of plasma cholinesterase could also be false positive for OP or CM exposure. It should be mentioned that the farmer’s baseline enzyme levels at the exposure-free period were not obtained because of the high frequency of spraying, and the reluctance of the farmers to donate blood.

The effects of pesticides on the immune system, either immunostimulation or immunosuppression, have been widely reported [5,11,28,29]. In the present study, immunological markers were assessed in serum/plasma and immune cells in order to determine the effects on both cellular and humoral immune responses. It was found that the farmer group showed significant reductions in both the percentage and the absolute number of CD19^+^cells. CD19^+^ cells are B lymphocytes that are involved in the humoral immune response. Garg *et al.* [13] showed a reduction in the number and function of B lymphocytes in chicks chronically exposed to small amounts of OP and synthetic pyrethroid pesticides. Down-regulation of the humoral immunity was also observed in chickens treated with carbendazim [30]. Many studies have observed reductions in the number of splenic plaque forming cells in experimental animals exposed to pesticide suggesting impaired humoral immunity [31,32]. The finding in the orchid farmers suggests that exposure to pesticide mixtures decreased the B lymphocytes in humans.

Although a reduction in the number of B lymphocytes in exposed farmers was detected in the present study, the concentration of globulin in serum and the levels of IgG, IgA and IgM were not significantly different from those of the controls. Information about the effect of pesticides exposure on the level of immunoglobulin is limited and controversial. Jablonicka *et al.* [33] studied workers occupationally exposed to the fungicide zinc manganese ethylenebisdithiocarbamate (mancozeb) showed an increase in serum IgG, IgE and β2-macroglobulin. Stiller-Winkler *et al.* [34] reported decreased serum IgM but not IgG and IgA in the sera of pesticide applicators, while Undeger and Basaran [35] detected no changes in serum IgG, IgA, IgM and C3 complement levels. Steerenberg *et al.* [11] reported increases in IgG4 and complement levels but a decrease in IgA in European pesticide workers. These discrepancies may be a result of differing effects of the individual components in the pesticide mixtures such that only certain subclasses of immunoglobulins were affected by each pesticide.

Unlike other immunoglobulin classes, it was shown here that there was a significant increase in total serum IgE in the orchid farmer group. An elevated IgE level, though not diagnostic of any single condition, is frequently observed in allergic and/or helminthic parasitic infections [36]. A study by Chatzi *et al.* [3] on grape farmers in Greece has found that occupational exposure to multiple agricultural chemicals could be related to allergic rhinitis. Fukuyama *et al.* [37] in a study using mouse model has demonstrated that low-dose of phenoxyacetic acid, organophosphorus, and carbamate pesticides can cause allergic reactions. It should be mentioned that the orchid farmers in this study did not show or report having any allergic reactions including asthma and allergic dermatitis. Unfortunately, the incidence of helminthic infections in this group of Thai orchid farmers was not studied.

This study did not observe significant changes in the levels of serum lysozyme and the functional immune response as assessed by lymphoproliferative activities to Con A and PWM. Thus these arms of defense were either unaffected, or that this was the overall outcome as a result of the redundancy and compensatory mechanisms of the immune system.

In addition to immunological parameters, the present study evaluated the hematological and biochemical status of the farmers. In general, there was no significant difference in these health profiles between the farmer and control groups. The parameter in the farmer group that showed a significant reduction was serum albumin, thus resulting in a significant reduction in total serum protein. However, the mean values of these parameters in both study groups were within the reference ranges. Albumin is an important serum protein and is synthesized by the liver. Its level in serum is dependent on a number of factors such as nutritional status, liver function, hormonal factors and urinary and gastrointestinal losses [38]. Since the primary indicators of the kidney function (BUN, creatinine) as well as liver function (AST, ALT, ALP) did not differ between both groups and their levels were within the normal ranges of reference values, the loss of albumin via the kidney or decreased albumin production by the liver as a result of liver cell death seemed unlikely. The lower serum albumin level in the farmers could result from nutritional status and/or the liver synthetic function. The farmers seemed to be healthy and probably not malnourished. The lower albumin level in the farmers might be the result of some subtle change in expression levels in the liver.

It should be mentioned that almost all of the farmers and controls were smokers and occasional/frequent alcohol drinkers. Many farmers believed that drinking a bottle of beer or carbonated soft drink after spraying could help excrete the toxicants. Since alcohol could exert hormetic effects on various biological systems [39], it is not certain whether this behavior of the farmers and controls had on the changes observed in this study. For the above mentioned reasons, analysis of the effects of these confounders could not be made with the present data.

This study had originally planned to measure the metabolites of the pesticides in the blood/urine of the farmers. However, due to the small research budget, limited access to the required equipment, *i.e.*, LC-MS-MS or GC-MS-MS and, admittedly, since it was not known at the time that the farmers suffered any ill effects from pesticide exposure, it was therefore decided to investigate whether the farmers had any biological alterations first. The analysis of metabolites would be the next logical step to follow.

Admittedly, this research was difficult to conduct due partly to time constraints and the reluctance of the farmers to participate in this study that resulted in a small sample size. The more difficult aspect was the inability to precisely know the type(s) of pesticide used and at what concentration, as well as the time after exposure for each farmer. Therefore, it was difficult to establish a causal relationship between pesticide exposure and changes in the immunological and biochemical parameters. This is in accordance with Corsini *et al.* [5] who mentioned that the assessment of toxicity of pesticides on the immune and probably other systems in agricultural workers co-exposed to several compounds was a very difficult task. In spite of these difficulties, the results obtained suggested that occupational exposure to cocktails of pesticides in these orchid farmers may be associated with some subtle changes in their immune systems (decreased B lymphocytes subpopulation and increased IgE level) and some biochemical parameters (reduction in serum albumin level). Since the farmers showed normal hematological parameters and liver and kidney functions and were in good general health, the alterations in the immunological and biochemical parameters observed were not likely to be of clinical significance. However, these alterations strongly indicated that more caution in handling pesticides by the orchid farmers is warranted.

## 5. Conclusions

Various studies have found that most Thai orchid farmers used excessive amounts of pesticides, but no toxicological study had been carried out on them. The present study found that these farmers, especially sprayers who frequently contacted with mixtures of pesticides, showed alterations of some immunological and biochemical parameters. Although these changes may not be clinically significant, they strongly indicate that the farmers should be more cautious in handling pesticides especially with regards to the use of correct and effective PPE.

## Abbreviations

ALT: alanine aminotransferase
ALP: alkaline phosphatase
AST: aspartate aminotransferase
BUN: blood urea nitrogen
CM: carbamate
Con A: concanavalin A
CV: coefficient of variation
MCH: mean corpuscular hemoglobin
MCHC: mean corpuscular hemoglobin concentration
MCV: mean corpuscular volume
MTT: 3-(4,5-dimethylthiazol-2-yl)-2,5-diphenyltetrazolium bromide
OP: organophosphate
PBMC: peripheral blood mononuclear cells
PPE: personal protective equipment
PWM: pokeweed mitogen
PY: pyrethroid

## References

[1] Summary of Imported Pesticides (2008–2012). http://www.oae.go.th/ewt_news.php?nid=146.

[2] Srivirojana N., Theptepa T., Punpuing S., Guest P., Khumtong T., Chankham O., Suvansrual A. Population pressure, utilization of chemicals in agriculture, health outcomes and solid waste management. Proceedings of the International Conference on Integrated Solid Waste Management in Southeast Asian Cities.

[3] Chatzi L., Alegakis A., Tzanakis N., Siafakas N., Kogevinas M., Lionis C. (2007). Association of allergic rhinitis with pesticide use among grape farmers in Crete, Greece. Occup. Environ. Med..

[4] (2014). Report on Prosecuted Cases of Adulteration of Agrochemicals Fiscal Year 2011–2014.

[5] Corsini E., Liesivuori J., Vergieva T., Van Loveren H., Colosio C. (2008). Effects of pesticide exposure on the human immune system. Hum. Exp. Toxicol..

[6] Karami-Mohajeri S., Abdollahi M. (2011). Toxic influence of organophosphate, carbamate, and organochlorine pesticides on cellular metabolism of lipids, proteins, and carbohydrates: A systematic review. Hum. Exp. Toxicol..

[7] Androutsopoulos V.P., Hernandez A.F., Liesivuori J., Tsatsakis A.M. (2013). A mechanistic overview of health associated effects of low levels of organochlorine and organophosphorous pesticides. Toxicology.

[8] Thrasher J.D., Madison R., Broughton A. (1993). Immunologic abnormalities in humans exposed to chlorpyrifos: Preliminary observations. Arch. Environ. Health.

[9] Hundekari I.A., Suryakar A.N., Rathi D.B. (2013). Acute organo-phosphorus pesticide poisoning in north Karnataka, India: Oxidative damage, haemoglobin level and total leukocyte. Afr. Health Sci..

[10] Hadnagy W., Leng G., Sugiri D., Ranft U., Idel H. (2003). Pyrethroids used indoors—Immune status of humans exposed to pyrethroids following a pest control operation—A one year follow-up study. Int. J. Hyg. Environ. Health.

[11] Steerenberg P., van Amelsvoort L., Colosio C., Corsini E., Fustinoni S., Vergieva T., Zaikov C., Pennanen S., Liesivuori J., Van Loveren H. (2008). Toxicological evaluation of the immune function of pesticide workers: A European wide assessment. Hum. Exp. Toxicol..

[12] Klucinski P., Kossmann S., Tustanowski J., Friedek D., Kaminska-Kolodziej B. (2001). Humoral and cellular immunity rates in chemical plant workers producing dust pesticides. Med. Sci. Monit..

[13] Garg U.K., Pal A.K., Jha G.J., Jadhao S.B. (2004). Haemato-biochemical and immuno-pathophysiological effects of chronic toxicity with synthetic pyrethroid, organophosphate and chlorinated pesticides in broiler chicks. Int. Immunopharmacol..

[14] Chatterjee S., Basak P., Chaklader M., Das P., Pereira J.A., Chaudhuri S., Law S. (2013). Pesticide induced marrow toxicity and effects on marrow cell population and on hematopoietic stroma. Exp. Toxicol. Pathol..

[15] Chatterjee S., Basak P., Chaklader M., Das P., Pereira J.A., Chaudhuri S., Law S. (2014). Pesticide induced alterations in marrow physiology and depletion of stem and stromal progenitor population: An experimental model to study the toxic effects of pesticide. Environ. Toxicol..

[16] Ellman G.L., Courtney K.D., Andres V., Feather-Stone R.M. (1961). A new and rapid colorimetric determination of acetylcholinesterase activity. Biochem. Pharmacol..

[17] Keller J.M., McClellan-Green P.D., Kucklick J.R., Keil D.E., Peden-Adams M.M. (2006). Effects of organochlorine contaminants on loggerhead sea turtle immunity: Comparison of a correlative field study and *in vitro* exposure experiments. Environ. Health Perspect..

[18] Ng S.P., Zelikoff J.T. (2008). The effects of prenatal exposure of mice to cigarette smoke on offspring immune parameters. J. Toxicol. Environ. Health A.

[19] Gonzalez-Quintela A., Alende R., Gude F., Campos J., Rey J., Meijide L.M., Fernandez-Merino C., Vidal C. (2008). Serum levels of immunoglobulins (IgG, IgA, IgM) in a general adult population and their relationship with alcohol consumption, smoking and common metabolic abnormalities. Clin. Exp. Immunol..

[20] Webster H.K., Pattanapanyasat K., Phanupak P., Wasi C., Chuenchitra C., Ybarra L., Buchner L. (1996). Lymphocyte immunophenotype reference ranges in healthy thai adults: Implications for management of HIV/AIDS in Thailand. Southeast. Asian J. Trop. Med. Public Health.

[21] Annual Epidemiological Surveillance Report 2008. http://www.boe.moph.go.th/Annual/Annual%202551/Part1_51/5551_PesticidePoisoning.doc.

[22] Bureau of Occupational and Environmental Diseases (2006). Healthy’s Farmer Project.

[23] Jintana S., Sming K., Krongtong Y., Thanyachai S. (2009). Cholinesterase activity, pesticide exposure and health impact in a population exposed to organophosphates. Int. Arch. Occup. Environ. Health.

[24] Kachaiyaphum P., Howteerakul N., Sujirarat D., Siri S., Suwannapong N. (2010). Serum cholinesterase levels of Thai chilli-farm workers exposed to chemical pesticides: Prevalence estimates and associated factors. J. Occup. Health.

[25] Eddleston M., Buckley N.A., Eyer P., Dawson A.H. (2008). Management of acute organophosphorus pesticide poisoning. Lancet.

[26] Sidell F.R., Kaminskis A. (1975). Temporal intrapersonal physiological variability of cholinesterase activity in human plasma and erythrocytes. Clin. Chem..

[27] Garabrant D.H., Aylward L.L., Berent S., Chen Q., Timchalk C., Burns C.J., Hays S.M., Albers J.W. (2009). Cholinesterase inhibition in chlorpyrifos workers: Characterization of biomarkers of exposure and response in relation to urinary TCPy. J. Expo. Sci. Environ. Epidemiol..

[28] Colosio C., Birindelli S., Corsini E., Galli C.L., Maroni M. (2005). Low level exposure to chemicals and immune system. Toxicol. Appl. Pharmacol..

[29] Colosio C., Corsini E., Barcellini W., Maroni M. (1999). Immune parameters in biological monitoring of pesticide exposure: Current knowledge and perspectives. Toxicol. Lett..

[30] Singhal L.K., Bagga S., Kumar R., Chauhan R.S. (2003). Down regulation of humoral immunity in chickens due to carbendazim. Toxicol. Vitro.

[31] Tamang R.K., Jha G.J., Gupta M.K., Chauhan H.V., Tiwary B.K. (1988). *In vivo* immunosuppression by synthetic pyrethroid (*Cypermethrin*) pesticide in mice and goats. Vet. Immunol. Immunopathol..

[32] Blakley B.R., Yole M.J., Brousseau P., Boermans H., Fournier M. (1999). Effect of chlorpyrifos on immune function in rats. Vet. Hum. Toxicol..

[33] Jablonicka A., Polakova H., Karelova J., Vargova M. (1989). Analysis of chromosome aberrations and sister-chromatid exchanges in peripheral blood lymphocytes of workers with occupational exposure to the mancozeb-containing fungicide novozir MN80. Mutat. Res..

[34] Stiller-Winkler R., Hadnagy W., Leng G., Straube E., Idel H. (1999). Immunological parameters in humans exposed to pesticides in the agricultural environment. Toxicol. Lett..

[35] Undeger U., Basaran N. (2001). Effects of pesticide exposure on serum immunoglobulin and complement levels. Immunopharmacol. Immunotoxicol..

[36] Dessaint J.P., Bout D., Wattre P., Capron A. (1975). Quantitative determination of specific IgE antibodies to *Echinococcus granulosu*s and IgE levels in sera from patients with hydatid disease. Immunology.

[37] Fukuyama T., Tajima Y., Ueda H., Hayashi K., Shutoh Y., Harada T., Kosaka T. (2009). Allergic reaction induced by dermal and/or respiratory exposure to low-dose phenoxyacetic acid, organophosphorus, and carbamate pesticides. Toxicology.

[38] Limdi J.K., Hyde G.M. (2003). Evaluation of abnormal liver function tests. Postgrad. Med. J..

[39] Romeo J., Warnberg J., Nova E., Diaz L.E., Gomez-Martinez S., Marcos A. (2007). Moderate alcohol consumption and the immune system: A review. Brit. J. Nutr..

